# Sex Differences in Shotgun Proteome Analyses for Chronic Oral Intake of Cadmium in Mice

**DOI:** 10.1371/journal.pone.0121819

**Published:** 2015-03-20

**Authors:** Yoshiharu Yamanobe, Noriyuki Nagahara, Takehisa Matsukawa, Takaaki Ito, Kanako Niimori-Kita, Momoko Chiba, Kazuhito Yokoyama, Toshihiro Takizawa

**Affiliations:** 1 Isotope Research Center, Nippon Medical School, Bunkyo-ku, Tokyo, Japan; 2 Department of Epidemiology and Environmental Health, Juntendo University, Faculty of Medicine, Bunkyo-ku, Tokyo, Japan; 3 Department of Pathology and Experimental Medicine, Kumamoto University Graduate School of Medical Sciences, Hongo, Kumamoto, Japan; University of Navarra School of Medicine and Center for Applied Medical Research (CIMA), SPAIN

## Abstract

Environmental diseases related to cadmium exposure primarily develop owing to industrial wastewater pollution and/or contaminated food. In regions with high cadmium exposure in Japan, cadmium accumulation occurs primarily in the kidneys of individuals who are exposed to the metal. In contrast, in the itai-itai disease outbreak that occurred in the Jinzu River basin in Toyama Prefecture in Japan, cadmium primarily accumulated in the liver. On the other hand, high concentration of cadmium caused renal tubular disorder and osteomalacia (multiple bone fracture), probably resulting from the renal tubular dysfunction and additional pathology. In this study, we aimed to establish a mouse model of chronic cadmium intake. We administered cadmium-containing drinking water (32 mg/l) to female and male mice *ad libitum* for 11 weeks. Metal analysis using inductively coupled plasma mass spectrometry revealed that cadmium accumulated in the kidneys (927 x 10 + 185 ng/g in females and 661 x 10 + 101 ng/g in males), liver (397 x 10 + 199 ng/g in females and 238 x 10 + 652 ng/g in males), and thyroid gland (293 + 93.7 ng/g in females and 129 + 72.7 ng/g in males) of mice. Female mice showed higher cadmium accumulation in the kidney, liver, and thyroid gland than males did (p = 0.00345, p = 0.00213, and p = 0.0331, respectively). Shotgun proteome analyses after chronic oral administration of cadmium revealed that protein levels of glutathione *S*-transferase Mu2, Mu4, and Mu7 decreased in the liver, and those of A1 and A2 decreased in the kidneys in both female and male mice.

## Introduction

Cadmium accumulates in humans because of long-term ingestion of cadmium-contaminated food and water. Environmental diseases that have occurred such as itai-itai disease in the Jinzu River basin in Toyama Prefecture in Japan were due to industrial wastewater pollution [1, 2], where is famous rice producing area. Cadmium was contained waste discharged from a mine industry. Local residents consume rice harvesting their own rice fields and used this river water as their drinking water. Therefore, local residents were exposed to much cadmium. Furthermore, there are several cadmium-polluted regions owing to natural soil contamination in Japan [3–5]. Itai-itai disease primarily occurred in post-menopausal women and in mainly characterized by renal tubular disorder, which is known as tubulopathy and osteomalacia accompanied with osteopolosis [6, 7]. In the case of severe renal tubular disorder cadmium cannot accumulate much more in the damaged kidney which has been confirmed by autopsy findings of itai-itai disease patients. In contrast, the liver was not affected and there was no histological change in the liver [7]. The same cadmium accumulation pattern as in itai-itai disease was observed in inhabitants of cadmium-polluted area in Nagasaki Prefecture in Japan [8]. For inhabitants in other cadmium-polluted area, therefore, the kidney was predominant organ accumulating cadmium [1–9]. It is generally considered that cadmium-induced osteomalacia resulting in multiple bone fractures [10–12] and anemia [13–15] were results of renal tubulopathy.

A mouse model of acute cadmium poisoning has been established [16], but no model for chronic cadmium exposure has been established. In this study, our intent was to assess sexual differences in tissue-specific cadmium accumulation in chronic oral cadmium exposure. Using mice for studies has several advantages including cost reduction and the ability to generate genetically modified mice including transgenic, gene-knockout, and gene-knockdown mice to help clarify the pathogenesis of human itai-itai disease. In contrast, genetically modified rats have not been developed, although a rat model of cadmium poisoning has been established [17, 18].

In previous autopsy cases of itai-itai disease [19], the liver, kidney, and thyroid gland showed significant levels of cadmium accumulation. After chronic oral administration of cadmium to mice, tissue-specific accumulation in these organs was investigated using inductively coupled plasma mass spectrometry (ICP-MS) and light microscopy. Since proteomic analysis has only been performed in acute cadmium administration (intraperitoneal) studies in mice [20, 21], we performed shotgun proteome analyses on the kidney and liver after chronic oral cadmium administration.

## Materials and Methods

### Ethics statement

All animal experiments were carried out in strict accordance with the recommendations in the Guide for the Care and Use of Laboratory Animals of the National Institutes of Health. The protocol was approved by the Committee on the Ethics of Animal Experiments of Nippon Medical School (Permit Number: 24-177). All surgery was performed under sodium pentobarbital anesthesia, and all efforts were made to minimize suffering.

### Animals

Nine-week-old C57BL/6NCrSlc male and female mice (Sankyo Laboratory Service Corporation, Inc., Tokyo, Japan) were used for these studies. They were divided into control and experimental groups (4 mice each) and maintained in a clean room at 24 ± 2.0°C, with humidity of 55 ± 5% under a 14-h diurnal lighting regimen (dark cycle; 2000 to 0600 hours).

### Chemicals

Cadmium chloride 2.5-hydrate (Wako Pure Chemicals. Osaka, Japan), diethyl ether (Wako Pure Chemicals. Osaka, Japan), nitric acid (ultrapure analytical grade reagent, Tama Chemical Co. Ltd. Kawasaki, Japan), and cadmium standard solution (Kanto Chemical Co. Ltd. Tokyo, Japan) were purchased. Bovine muscle (RM-8414, National Institute of Standards and Technology, Maryland, USA) and bovine liver (BCR-185, Institute for Reference Materials and Measurements, Geel, Belgium) were used as reference materials. Other chemicals were of analytical grade.

### Experimental protocol

The cadmium dose administered was 6% of the LD_50_ for mice (60 mg/kg/day [22, 23]). The average body weight was estimated to be 35 g throughout the study and was used to calculate the cadmium dose to be administered (126 μg/day/mouse). The average volume of drinking water was estimated to be 4 ml/day/mouse, based on our previous experiments [24]. The cadmium concentration of the solution was 32 mg/l (64 mg of cadmium chloride 2.5-hydrate in Milli-Q water).

For the experimental group, the solution was administered *ad libitum* for 11 weeks in the drinking water supplied. The solution was changed once a week. The mice were fed sterilized commercial rodent pellets containing less than 0.06 μg/μl cadmium (MF, Oriental Yeast Co. Tokyo, Japan) *ad libitum*. For the control group, Milli-Q water and the same rodent pellets were given *ad libitum*. In the 11th week of the study, the mice were anesthetized with sodium pentobarbital, and perfused with cold 5% glucose solution through the inferior vena cava and the left ventricle of the heart. The thyroid gland, liver, and kidney were then excised.

### Microscopic examination

Harvested organs were fixed with phosphate (50 mM)-buffered 10% formaldehyde (pH 7.3) and stored at room temperature. The organs were paraffin-embedded and sections were stained with hematoxylin-eosin.

### Metal analysis

Harvested organ samples were frozen at −80°C until analysis. Each sample as digested with 400 μl of nitric acid (Tamapure AA-100, ultra-high-purity-grade, Tama Chemicals, Kawasaki, Japan) overnight, and then 200 μl of hydrogen peroxide was added. Each sample was then microwaved (MLS-1200 MEGA, Milestone General, K.K., Kawasaki, Japan). The amount of cadmium in each sample was measured using ICP-MS (ELAN DRC-II, PerkinElmer Japan Inc. Tokyo, Japan). Tissue cadmium content represents ng/g wet tissue weight.

### Calibration curve for cadmium ion determination

Cadmium ions (3.125, 6.25, 12.5, 5, 50, and 100 ng/ml) in 5% nitric acid were used as a standard cadmium solution. The calibration curve obtained using ICP-MS was linear.

### Sample preparation for proteomic analysis

The liver and kidney were used for gunshot proteome analyses. Organs mixed with equal amount of the liver or kidney from four mice in each control and experimental group were combined and homogenized in a 10 x volume of UPX solution (UPX^TM^ Universal Protein Extraction Kit, Expedeon, San Diego, CA, USA) containing protease inhibitor (cOmplete Mini, Roche Diagnostics Japan, Tokyo, Japan) and phosphatase inhibitor (PhosSTOP, Roche Diagnostics Japan, Tokyo, Japan) with ULTRA TRRUX (T10, IKA Japan, Osaka, Japan). Each homogenate was incubated for 5 min at 100°C and was centrifuged at 15,000 x g for 10 min at 4°C.

Methyl alcohol (400 μl) was added to 100 μl of each supernatant and mixed using a vortex mixer. Chloroform (100 μl) was added to the mixture and mixed by vortexing. Milli-Q water (300 μl) was added to the mixture and vortexed. The mixture was centrifuged at 1,500 x g for 5 min at 4°C. The proteins were extracted into the interface between the organic and aqueous layers, and the upper layer was removed. Methyl alcohol (400 μl) was added to the tube and the mixture was shaken by top-bottom inversion. Then, methyl alcohol was removed and the precipitate was dried using SpeedVac (SAVANT DNA120, Thermo Scientific Japan, Yokohama, Japan).

Each protein precipitate was dissolved in 20 μl of 8 M urea, and the volume was adjusted to 100 μl with 100 mM ammonium hydrogen carbonate. Then, 5 μl of 90 mM ammonium hydrogen carbonate containing 100 mM dithiothreitol and 10% acetonitrile was added to the mixture, and the mixture was incubated for 60 min at 37°C. After centrifugation at 15,000 x g for 5 min at 4°C, 100 μl of 90 mM ammonium hydrogen carbonate containing 100 mM iodoacetamide and 10% acetonitrile was added to the mixture. Carbamide methylation was performed by incubation in the dark for 30 min at 37°C. Then, 10 μl of 200 ng/μl trypsin (Promega KK, Tokyo, Japan) was added to the mixture and proteolysis was performed for 16 h at 37°C.

After the volume of the mixture was reduced to approximately 10 μl by using SpeedVac, 100 μl of solution A (0.1% trichloroacetic acid and 2% acetonitrile) was added to the mixture. For desalination, the mixture was applied to a C-TIP column (KT200, AMR, Tokyo, Japan) and the column was rinsed with 100 μl of solution A. Peptides were eluted with 100 μl of solution containing 0.5% trichloroacetic acid and 80% acetonitrile. The sample volume was reduced to approximately 5 μl by using SpeedVac and was adjusted to 20 μl with solution A.

### Mass spectrometry

Samples were analyzed using a mass spectrometer (amaZonTM ETD, Bruker Daltonics, Billerica, MA, USA) equipped with CaptiveSpray NSI source (Bruker Daltonics) via an HTS-PAL auto sampler (CTC Analysis, Zwingen, Switzerland). A nano-flow liquid chromatography system (Advance nanoLC, Michrom Bioresources Inc., Auburn, CA, USA) was equipped with a reverse-phase capillary column (Zaplous column C18, 0.1 mm × 150 mm, AMR Inc., Tokyo, Japan). A linear elution gradient from 6.4% acetonitrile with 0.1% formic acid to 41.6% acetonitrile with 0.1% formic acid was carried out for 120 min. The scan ranges were 300–1500 m/z and 100–2500 m/z for monovalent and divalent ions, respectively.

### Data analysis

After conversion of the raw ion trap tandem mass spectrometry (MS/MS) spectra data to the Mascot Generic format (MGF) by using DataAnalysis 4.0 (Bruker Daltonics), data sets were searched using a database (taxonomy: *Mus musculus*, peptide charges: 2+, 3+, and 4+, peptide tolerance: ± 2.0 Da, MS/MS tolerance: ± 0.8 Da, missed cleavages: 2, fixed modifications: carbamidomethyl cysteine). Proteins were validated and quantified using ProteoIQ software (ver. 2.7.1, PREMIER Biosoft International, Palo Alto, CA, USA).

### Statistical analysis

Differences between female and male mice were analyzed by unpaired Student’s *t* test using Excel (Microsoft, Redmond, WA, USA) and ANOVA using StatPlus ver. 5 (AnalystSoft Inc., Alexandria, VA, USA). All values are presented as mean ± standard deviation. A p value of less than 0.0500 was considered to indicate statistical significance.

## Results

### Cadmium administration and general conditions of mice

Water consumption for female and male mice was 3.4 and 3.6 ml/day/mouse, respectively, and the daily average dose of cadmium exposure was 108.1 μg and 112.1 μg/mouse for female and male mice, respectively. No animals died during the study nor were there any changes in the general condition of the animals in any group during the experimental period.

### Cadmium analysis

Cadmium levels in the liver, kidney, and thyroid gland are shown in Table 1. A two-way ANOVA demonstrated an interaction between sex difference and cadmium exposure in cadmium storage in the liver, kidney, and thyroid gland (p = 0.000550 (F = 21.7), p = 0.000250 (F = 26.4), and p = 0.0199 (F = 7.20), respectively. It is concluded that cadmium is significantly accumulated in female more than male mice. Further statistical analyses (*t*-tests) among each groups indicated that, in the female control group, cadmium levels in the kidney and thyroid gland were significantly higher than those in the male group (p = 0.00155, and p = 0.000324, respectively, Table 1). Liver cadmium levels were not significantly different between female and male groups (p = 0.0581) (Table 1). Ratios in cadmium accumulation (ng/g tissue) in the liver and kidney to that of the thyroid gland were 1.8 and 3.5, respectively in female mice and 4.4 and 10.7, respectively in male mice. These findings confirmed that cadmium accumulated in various tissues after daily ingestion of trace-contaminated rodent pellets and water, and the kidney is a primary organ for cadmium accumulation in a trace-contaminated environment.

**Table 1:** Cadmium contents in tissues.

	Female		Male	
Tissue	Control (ng/g)	Experimental (ng/g)	Control (ng/g)	Experimental (ng/g)
Liver	9.39 ± 2.23 ^a^	397 x 10 ± 199 ^b^	6.06 ± 1.74 ^a^	238 x 10 ± 652 ^b^
Kidney	18.4 ± 4.01 ^c^	927 x 10 ± 185 ^d^	14.7 ± 2.38 ^c^	(661 ± 101) x10 ^d^
Thyroid gland	5.32 ± 1.61 ^e^	293 ± 93.7 ^f^	1.37 ± 0.386 ^e^	129 ± 72.7 ^f^

Sexual differences in cadmium contents in the liver, the kidney, and the thyroid gland between female and male control groups

^a^ p = 0.0581

^b^ p = 0.00343; and

^c^ p = 0.00155, respectively and between female and male experimental groups

^d^ p = 0.00213

^e^ p = 0.000324; and

^f^ p = 0.0331, respectively.

Data are shown as the mean ± S.D., (n = 4). ANOVA results are described in the text.

After chronic oral administration of cadmium, the kidney, liver and thyroid gland significantly accumulated the metal both in female and male mice, and the accumulation ratios for these organs were 15.6:36.5:1.0 in female mice, respectively and 14.2:39.4:1.0 in male mice, respectively (Table 1). In the female experimental group, cadmium contents in the liver, kidney, and thyroid gland were significantly higher than in male group (p = 0.00343, p = 0.00213, and p = 0.0331, respectively, Table 1). These findings show that the kidney is a primary organ for cadmium accumulation by chronic oral administration.

### Microscopic examination

**Fig 1:**
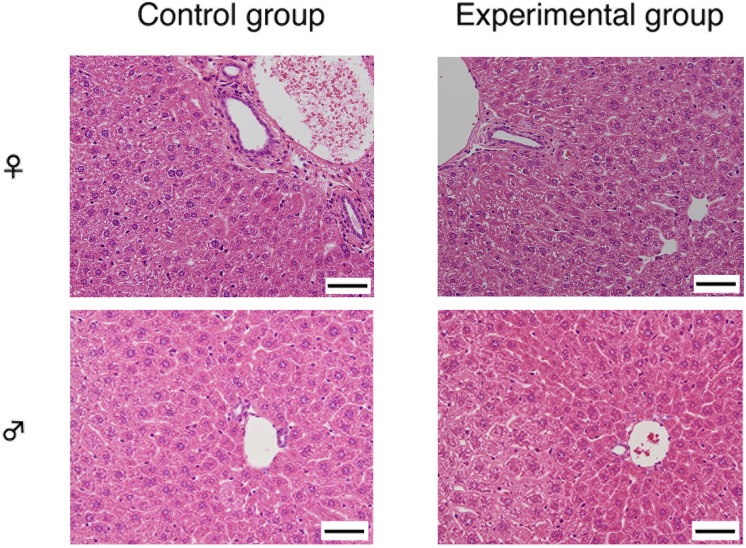
Microscopic examination of the liver after oral chronic administration of cadmium. Left side; control group, right side; experimental group, upper side; female mice, and lower side; male mice. Scale bar is 50 μm.

**Fig 2:**
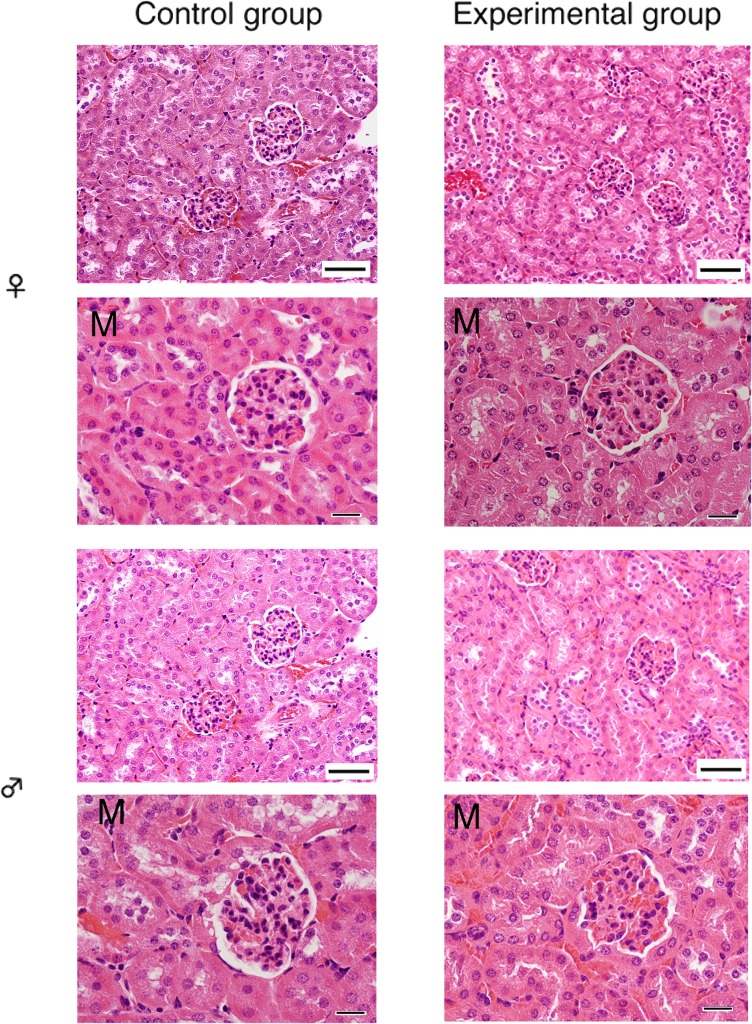
Microscopic examination of the kidney after oral chronic administration of cadmium. Left side; control group, right side; experimental group, upper side; female mice, and lower side; male mice; M, magnified microscopic views. Thick and thin scale bars are 50 and 20 μm, respectively.

**Fig 3:**
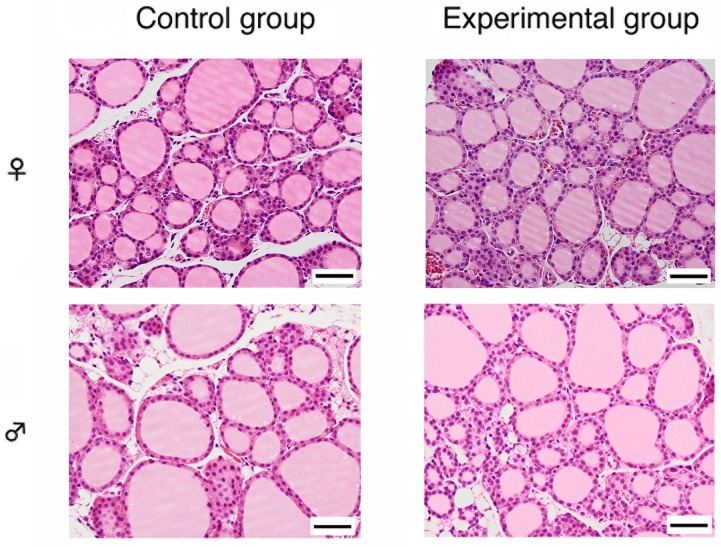
Microscopic examination of the thyroid gland after oral chronic administration of cadmium. Left side; control group, right side; experimental group, upper side; female mice, and lower side; male mice. Scale bar is 50 μm.

There were no significant microscopic changes of hepatocytes in the liver (Fig. 1), renal tubular cells in the kidney (Fig. 2), and follicular epithelial cells in the thyroid gland (Fig. 3) after cadmium administration.

### Proteomic analysis

The numbers of proteins identified in female mice were 226 and 238 for the liver in the control (Table 2) and treated (Table 3) groups, respectively, and the corresponding numbers of the kidney were 183 (Table 4) and 201 (Table 5). In male mice, the numbers of proteins identified were 240 and 235 for the liver in the control (Table 6) and treated (Table 7) groups, respectively, and the corresponding numbers for the kidney were 248 (Table 8) and 202 (Table 9).

**Table 2:** List of proteomic analysis for the control liver in female group.

Sequence name	Peptide number	Probability
myosin light chain 1/3, skeletal muscle isoform isoform 3 & 1f	4	1
tropomyosin beta chain	3	1
**aldehyde dehydrogenase X, mitochondrial pre**	**3**	**0.93**
tropomyosin alpha-1 chain isoform 1∼10	3	0.94
eukaryotic initiation factor 4A-I isoform −2	2	1
retinal dehydrogenase 2	2	1
lysosomal acid lipase/cholesteryl ester hydrolase pre	2	1
inorganic pyrophosphatase	2	1
**glutathione S-transferase Mu2, Mu4 (isoform 1 & 2) & Mu7**	**2**	**1**
malete dehydrogenase, cytoplasmic	2	1
retinal dehydrogenase 2	2	1
aldehyde dehydrogenase, cytosolic 1	2	1
**adenosylhomocysteinase**	**2**	**1**
inorganic pyrophosphatase	2	0.95
beta-enolase isoform 1 & 2	1	1
T-complex protein 1 subunit zeta	1	1
**cytochrome P450 2C37**	**1**	**1**
fatty acid-binding protein, heart	1	1
glutathione S-transferase Mu 3 & 5	1	1
voltage-dependent anion-selective channel protein 3	1	1
peroxisomal acyl-coenzyme A oxidase 1	1	1
myosin regulatory light chain 2, skeletal muscle isoform	1	1
very long-chain specific acyl-CoA dehydrogenase, mitochondrial pre	1	1
protein DJ-1	1	1
apolipoprotein C-III pre	1	1
small glutamine-rich tetratricopeptide repeat-containing protein alpha	1	1
**ras GTPase-activating-like protein IQGAP2**	**1**	**1**
methylcrotonoyl-CoA carboxylase beta chain, mitochondria	1	1
argininosuccinate lyase	1	1
glial fibrillary acidic protein isoform 1 & 2	1	0.99
annexin A2	1	0.99
ctyochrome 0459 2D10	1	0.99
myosin light chain 3 & 4	1	0.99
elongation factor Tu, mitochondrial isoform 1	1	0.99
cathepsin D pre	1	0.99
glutathione S-transferase A4	1	0.98
eukaryotic translation initiation factor 1tacking protein 2	1	0.95
isochorismatase domain-containing protein 2A, mitochondrial pre	1	0.93
selenocysteine lyase	1	0.93
myoglobin	1	0.88

Top 57 proteins exhibiting the probability of more than 0.8 are shown (17 family proteins are included). Proteins in bold letters were observed in the control liver in male group. Pre; precursor.

**Table 3:** List of proteomic analysis for the experimental liver in female group.

Sequence name	Peptide number	Probability
selenium-binding protein	3	1
histone H2B family	3	1
H2b histone family, member A	3	1
hydroxymethylglutaryl-CoA synthase, mitochondrial pre	2	1
isocitrate dehydrogenase (NADP) cytoplasmic	2	1
ferritin light chain 1 & 2	2	1
alpha-actinin-1 & 4	2	1
cytochrome P450 2F2	2	0.9
peptidyl-prolyl cis-trans isomerase H (isoform 1& 2) & C	1	1
histone H2B type 1-A	1	1
alpha-1-antitrypsin 1–5 pre	1	1
tubulin alpha-3 chain	1	1
eukaryotic translation initiation factor 3 subunit F	1	1
UDP-glucuronosyltransferase 1–2, 1–6, 1–7C & 1–9 pre	1	1
UDP glycosyltransferase 1 family	1	1
hydroxymethylglutaryl-CoA lyase, mitochondrial pre	1	1
prohibitin	1	1
protein disulfide-isomerase A6	1	1
ras-related protein Rap-1a & 1b pre	1	1
peroxisomal membrane protein	1	1
protein dpy-30 homolog	1	1
UTP—glucose-1-phosphate uridylyltransferase	1	1
transitional endoplasmic reticulum ATPase	1	1
40S ribosomal protein S10	1	1
T-complex protein 1 subunit delta	1	1
carboxylesterase 3 pre	1	1
ornithine aminotransferase, mitochondrial pre	1	1
mitochondrial-processing peptidase subunit alpha pre	1	0.99
MOSC domain-containing protein 2, mitochondrial pre	1	0.99
NADH dehydrogenase subunit 4	1	0.99
UDP-glucuronosyltransferase 2B17 pre	1	0.99
UDP glucuronosyltransferase 2 family, polypeptide B37	1	0.99
**secernin-2**	**1**	**0.98**
cytochrome b5	1	0.98
beta-galactosidase pre	1	0.98
NADH dehydrogenase 1 alpha subcomplex subunit 10, mitochondrial pre	1	0.98
calcium-binding mitochondrial carrier protein Aralar2 isoform 1 & 2	1	0.98
short-chain specific acyl-CoA dehydrogenase, mitochondrial pre	1	0.98
thioredoxin-dependent peroxide reductase, mitochondrial pre	1	0.97
**peroxisomal sarcosine oxidase**	**1**	**0.97**

Top 49 proteins exhibiting the probability of more than 0.8 are shown (9 family proteins are included). Proteins in bold letters were observed in the experimental liver in male group. Pre; precursor.

**Table 4:** List of proteomic analysis for the control kidney in female group.

Sequence name	Peptide number	Probability
argininosuccinate synthase	3	1
sodium/potassium-transporting ATPase subunit alpha-1 pre	3	0.99
alpha-actinin-4	2	1
isovaleryl-CoA dehydrogenase, mitochondrial pre	2	1
14-3-3 protein zeta/delta	2	1
hypthetical protein	1	1
**glutathione S-transferase A1 & A2**	**1**	**1**
alpha-actinin-1 & 3	1	1
thiopurine S-methyltransferase	1	1
**major urinary protein 2 pre**	**1**	**1**
major urinary protein 2-like	1	1
**major urinary protein 1 isoform a, b & c**	**1**	**1**
**major urinary protein 7, 11, 13 & 17**	**1**	**1**
NADH dehydrogenase (ubiquinone) flavoprotein 3, mitochondrial isoform 1 & 2	1	1
argininosuccinate lyase	1	1
elongation factor 2	1	1
sodium/potassium-transporting ATPase subunit alpha-1 pre	1	1
thioredoxin-dependent peroxide reductase, mitochondrial pre	1	0.99
2,4-dienoyl-CoA reductase, mitochondrial pre	1	0.99
ras-related protein Rap-1A & ab pre	1	0.99
alpha-aminoadipic semialdehyde synthase, mitochondrial pre	1	0.99
trifunctional enzyme subunit beta, mitochondrial pre	1	0.99
mitochondrial-processing peptidase subunit alpha pre	1	0.97
aldehyde dehydrogenase family 8 member A1	1	0.97
4F2 cell-surface antigen heavy chain isoform a & b	1	0.9
eukaryotic initiation factor 4A-I isoform 1 & 2	1	0.85
cytoplasmic dynein 1 light intermediate chain 1	1	0.82
peroxiredoxin-1	1	0.8
hypothetical protein LOC72371 isoform 1	1	0.8

Proteins exhibiting the probability of more than 0.8 are shown (10 family proteins are included). Proteins in bold letters were observed in the control kidney in male group. Pre; precursor.

**Table 5:** List of proteomic analysis for the experimental kidney in female group.

Sequence name	Peptide number	Probability
L-lactate dehydrogenase A chain isoform 1 & 2	2	1
selenium-binding protein 1	2	1
phosphoglycerate kinase 1	2	1
2-hydroxyacyl-CoA lyase 1	2	0.88
acetyl-CoA acetyltransferase, mitochondrial pre	2	0.85
14-3-3 protein theta	1	1
3-ketoacyl-CoA thiolase A & B, peroxisomal pre	1	1
78 kDa glucose-regulated protein pre	1	1
adenylyl cyclase-associated protein 1	1	1
ATPase family AAA domain-containing protein 1	1	1
coactosin-like protein	1	1
**cytochrome c oxidase subunit 6A1, mitochondrial**	**1**	**1**
eukaryotic translation initiation factor 1	1	1
glutathione S-transferase Mu 1 & 3	1	1
hemoglobin subunit zeta	1	1
histone H2B type 1-A	1	1
isocitrate dehydrogenase (NADP))cytoplasmic	1	1
phosphoglycerate kinase 2	1	1
phosphotriesterase-related protein	1	1
polyadenylate-binding protein 1	1	1
polyadenylate-binding protein 4 isoform 1 & 2	1	1
spectrin beta chain, brain 1 isoform 1 & 2	1	1
**transthyretin**	**1**	**1**
tubulin beta-2A & 2B chain	1	1
A-kinase anchor protein 1, mitochondrial	1	0.99
**cytochrome b-c1 complex subunit 2, mitochondrial pre**	**1**	**0.99**
extracellular superoxide dismutase pre	1	0.99
peptidyl-prolyl cis-trans isomerase B pre	1	0.99
alpha-1-antitrypsin 1–5 pre	1	0.98
alpha-crystallin B chain	1	0.98
enoyl-CoA hydratase domain-containing protein 2, mitochondrial pre	1	0.98
NADH dehydrogenase (ubiquinone) 1 alpha subcomplex subunit 10, mitochondrial pre	1	0.98
aldehyde dehydrogenase, mitochondrial pre	1	0.97
sulfite oxidase, mitochondrial pre	1	0.97
tropomyosin alpha-4 chain	1	0.96
catalase	1	0.95
low-density lipoprotein receptor-related protein 2 pre	1	0.92
3-hydroxyacyl-CoA dehydrogenase type-2	1	0.88
secretogranin-1 pre	1	0.88
catenin alpha-3 isoform 1 & 3	1	0.88

Top 47 proteins exhibiting the probability of more than 0.8 are shown (7 family proteins are included). Proteins in bold letters were observed in the experimental kidney in male group. Pre; precursor.

**Table 6:** List of proteomic analysis for the control liver in male group.

Sequence name	Peptide number	Probability
carbonic anhydrase 3	5	1
glycogen phosphorylase, liver form	5	1
iver carboxylesterase 31 isoform 1 & 2 pre	4	1
cytochrome P450 2F2	4	1
sarcosine dehydrogenase, mitochondrial pre	3	1
fatty acid synthase	3	1
elongation factor 2	3	1
hydroxymethylglutaryl-CoA synthase, mitochondrial pre	3	1
glutathione S-transferase Mu 1	3	1
arginase-1	3	1
alcohol dehydrogenase 1	3	1
cytochrome P450 2E1	3	1
aldehyde dehydrogenase, mitochondrial pre	3	1
sorbitol dehydrogenase	3	1
acyl-coenzyme A synthetase ACSM1, mitochondrial	3	1
**adenosylhomocysteinase**	**3**	**0.97**
parathymosin	2	1
bile acyl-CoA synthetase	2	1
**glutathione S-transferase Mu2, Mu4 & Mu7**	**2**	**1**
glycine N-methyltransferase	2	1
interferon-inducible GTPase 1	2	1
uricase	2	1
ATP-citrate synthase	2	1
estradiol 17 beta-dehydrogenase 5	2	1
ornithine aminotransferase, mitochondrial pre	2	1
protein disulfide-isomerase A6	2	1
UDP-glucuronosyltransferase 2B17 pre	2	1
**ras GTPase-activating-like protein IQGAP2**	**2**	**0.99**
**cytochrome P450 2C37**	**2**	**0.97**
transmembrane protein 135	2	0.88
cytochrome P450 2C50 isoform 1 & 2	2	0.8
hydroxymethylglutaryl-CoA synthase, cytoplasmic	1	1
glutathione S-transferase Mu 3	1	1
beta-enolase isoform 1 & 2	1	1
phosphoglycerate kinase 1	1	1
retinal dehydrogenase 2	1	1
**aldehyde dehydrogenase X, mitochondrial pre**	**1**	**1**
aldehyde dehydrogenase family 1 member A3	1	1
dehydrogenase/reductase SDR family member 1	1	1
hypothetical protein LOC70861	1	1

Top 45 proteins exhibiting the probability of more than 0.8 are shown (5 family proteins are included). Proteins in bold letters were observed in the control liver in female group. Pre; precursor.

**Table 7:** List of proteomic analysis for the experimental liver in male group.

Sequence name	Peptide number	Probability
actin, aortic smooth muscle	6	1
actin, gamma-enteric smooth muscle	6	1
Na(+)/H(+) exchange regulatory cofactor NHE-RF1	5	1
meprin A subunit alpha	4	1
triosephosphate isomerase	4	1
histone H4	4	0.92
beta-actin-like protein 2	3	1
acyl-coenzyme A synthetase ACSM2, mitochondrial isoform 1	3	1
L-lactate dehydrogenase B chain	3	1
ribonuclease UK114	3	0.84
acyl-CoA-binding protein isoform 1 & 2	2	1
angiotensin-converting enzyme	2	1
cytochrome c, somatic	2	1
aconitate hydratase, mitochondrial pre	2	1
sodium/potassium-transporting ATPase subunit alpha-1 pre	2	1
tropomyosin alpha-3 chain	2	1
cadherin-16 pre	2	1
complement component 1 Q subcomponent-binding protein	2	1
NADH dehydrogenase (ubiquinone) iron-sulfur protein 8, mitochondrial pre	2	1
tropomyosin alpha-1 chain isoform (1–10)	2	1
ezrin	2	1
ATP synthase subunit delta, mitochondrial pre	2	1
cytochrome P450 4B1	2	1
isocitrate dehydrogenase (NADP), mitochondrial pre	2	0.99
hypothetical protein LOC70950	2	0.94
L-lactate dehydrogenase C chain	2	0.88
apolipoprotein A-II pre	2	0.85
myosin light polypeptide 6	2	0.8
hypothetical protein LOC72097	1	1
V-type proton ATPase subunit F	1	1
histone H2A type 2–8	1	1
kidney androgen-regulated protein pre	1	1
tropomyosin beta chain	1	1
low-density lipoprotein receptor-related protein 2 pre	1	1
potassium-transporting ATPase alpha chain 1	1	1
sodium/potassium-transporting ATPase subunit alpha-2 & -3	1	1
histone H3.3	1	1
calbindin	1	1
angiotensin-converting enzyme isoform 2	1	1
alcohol dehydrogenase	1	1
**secernin-2** ^**a**^	**1**	**0.99**
**peroxisomal sarcosine oxidase** ^**a**^	**1**	**0.86**

Top 58 and ^a^ additional two proteins exhibiting the probability of more than 0.8 are shown (18 family proteins are included). Proteins in bold letters were observed in the experimental liver in female group. Pre; precursor.

**Table 8:** List of proteomic analysis for the control kidney in male group.

Sequence name	Peptide number	Probability
carbamoyl-phosphate synthase (ammonia), mitochondrial pre	19	1
fatty acid-binding protein, liver	9	1
carbonic anhydrase 3	6	1
arginase-1	5	1
78 kDa glucose-regulated protein pre	5	1
regucalcin	5	1
glycine N-methyltransferase	4	1
3-ketoacyl-CoA thiolase A, peroxisomal pre	4	1
peroxiredoxin-6	4	1
glutathione S-transferase A3	4	1
calreticulin pre	4	1
adenosylhomocysteinase	4	1
betaine—homocysteine S-methyltransferase 1	3	1
liver carboxylesterase 31-like isoform 1 & 2	3	1
glycogen phosphorylase, liver form	3	1
**major urinary protein 1 & 2 pre**	**3**	**1**
**major urinary protein 10, 11 & 17**	**3**	**1**
**major urinary protein 1 isoform b**	**3**	**1**
elongation factor 2	3	1
uricase	3	1
fatty acid synthase	2	1
cytochrome P450 2E1	2	1
liver carboxylesterase 22 pre	3	1
homogentisate 1,2-dioxygenase	3	1
alcohol dehydrogenase 1	3	1
glutamate dehydrogenase 1, mitochondrial pre	3	1
liver carboxylesterase 31 isoform 1 & 2 pre	2	1
**major urinary protein 7**	**2**	**1**
**major urinary protein 1 isoform a**	**2**	**1**
protein NDRG2 isoform 1 & 2	2	1
bile acyl-CoA synthetase	2	1
**glutathione S-transferase A1 & A2**	**2**	**1**
interferon-inducible GTPase 1	2	1
3-hydroxyanthranilate 3,4-dioxygenase	2	1
inorganic pyrophosphatase	2	1
2-hydroxyacyl-CoA lyase 1	2	1
long-chain-fatty-acid—CoA ligase 1	2	1
heterogeneous nuclear ribonucleoprotein K	2	1
glucokinase regulatory protein	2	1
lysosomal acid lipase/cholesteryl ester hydrolase pre	2	0.99

Top 47 proteins exhibiting the probability of more than 0.8 are shown (7 family proteins are included). Proteins in bold letters were observed in the control kidney in female group. Pre; precursor.

**Table 9:** List of proteomic analysis for the experimental kidney in male group.

Sequence name	Peptide number	Probability
phosphatidylethanolamine-binding protein 1	4	1
meprin A subunit alpha	4	1
medium-chain specific acyl-CoA dehydrogenase, mitochondrial	4	1
peroxiredoxin-5, mitochondrial pre	4	1
acyl-coenzyme A synthetase ACSM2, mitochondrial isoform 1	3	1
beta-actin-like protein 2	2	1
cadherin-16 precursor	2	1
sodium/potassium-transporting ATPase subunit alpha-1, 2 & 3 pre	2	1
fatty acid-binding protein, heart	2	1
cytochrome P450 4B1	2	1
L-lactate dehydrogenase B chain	2	1
solute carrier family 3, member 1	2	0.99
angiotensin-converting enzyme isoform 1	2	0.99
very long-chain specific acyl-CoA dehydrogenase, mitochondrial	2	0.98
prothymosin alpha	2	0.94
alcohol dehydrogenase	2	0.94
tropomyosin alpha-1 chain isoform 1–10	2	0.79
myosin light polypeptide 6	2	0.86
3,2-trans-enoyl-CoA isomerase, mitochondrial pre	1	1
actin-related protein 3 & 3B	1	1
annexin A2	1	1
**cytochrome b-c1 complex subunit 2, mitochondrial pre**	**1**	**1**
ezrin	1	1
glutathione reductase, mitochondrial pre	1	1
phosphoglycerate kinase 1	1	1
potassium-transporting ATPase alpha chain 1	1	1
protein S100-G	1	1
TOM1-like protein 2 isoform a, b & c	1	1
voltage-dependent anion-selective channel protein 3	1	1
WD repeat-containing protein 61 isoform a & b	1	1
angiotensin-converting enzyme isoform 2	1	1
citrate lyase subunit beta-like protein, mitochondrial pre	1	0.99
indolethylamine N-methyltransferase	1	0.99
nucleobindin-1 isoform 1 & 2	1	0.99
radixin isoform a	1	0.99
talin-1	1	0.99
**cytochrome c oxidase subunit 6A1, mitochondria**	**1**	**0.96**
glycerol kinase isoform 1 & 2	1	0.96
**transthyretin**	**1**	**0.96**
EMILIN-1 pre	1	0.95

Top 57 proteins exhibiting the probability of more than 0.8 are shown (17 family proteins are included). Proteins in bold letters were observed in the experimental kidney in female group. Pre; precursor.

The female hepatic proteins exhibiting the probability of more than 0.8 in control and experimental groups, were shown in Tables 2 and 3, respectively. Proteins expressed only in control livers were down-regulated or eliminated by chronic cadmium administration. It is noteworthy that seven proteins (Table 10) (proteins in bold letters in Table 2) including glutathione *S*-transferase Mu2, Mu7, and Mu7 were commonly identified in the livers of male mice in the control group (data shown below). On the other hand, proteins expressed only in the experimental treatment groups were up-regulated by chronic cadmium administration (Table 11). Two proteins, secernin-2 and peroxisomal sarcosine oxidase (Table 11) (proteins in bold letters in Table 3) were identical in the livers of the male mice in the experimental treatment groups (data shown below).

**Table 10:** Common down-regulated proteins in the liver and kidney after cadmium exposure.

Liver	Kidney
aldehyde dehydrogenase X, mitochondrial pre	glutathione S-transferase A1 & A2
glutathione S-transferase Mu2, Mu4 (isoform 1 & 2) & Mu7	major urinary protein 2-like
adenosylhomocysteinase	major urinary protein 1 isoform a, b & c
cytochrome P450 2C37	major urinary protein 7, 11, 13 & 17
ras GTPase-activating-like protein IQGAP2	

Proteins exhibiting the probability of more than 0.8 are shown. Pre; precursor.

**Table 11:** Common up-regulated proteins in the liver and kidney after cadmium exposure.

Liver	Kidney
secernin-2	cytochrome c oxidase subunit 6A1, mitochondrial
peroxisomal sarcosine oxidase	transthyretin
	cytochrome b-c1 complex subunit 2, mitochondrial pre

Proteins exhibiting the probability of more than 0.8 are shown. Pre; precursor.

The female renal proteins exhibiting the probability of more than 0.8 in control and experimental treatment groups were shown in Tables 4 and 5, respectively. In control kidneys, glutathione *S*-transferase A1 and A2 and major urinary protein (1 isoform a and b, 2 precursor, 7, 11, and 17) were identified (Table 10) (proteins in bold letters in Table 4), and were identical in the control kidneys in male mice (data shown below). In experimental treatment kidneys, three proteins (cytochrome c oxidase subunit 6A1 (mitochondrial form), transthyretin, cytochrome b-c1 complex subunit 2 (mitochondrial form precursor): (Table 11) (proteins in bold letters in Table 5) were up-regulated, which were identical to the control kidneys from male mice (data shown below).

The male hepatic proteins exhibiting the probability of more than 0.8 in proteomic analysis in control and experimental treatment groups were shown in Tables 6 and 7, respectively. Seven proteins (Table 10) (proteins in bold letters in Table 8), including glutathione *S*-transferase, Mu2, Mu4, and Mu7, were identified in the livers of female mice in the control group (Table 2). Two proteins (Table 11) (proteins in bold letters in Table 7) in the experimental treatment groups were up-regulated, which are identical to those in kidneys of male mice in the experimental treatment group (Table 5).

The male renal proteins exhibiting the probability of more than 0.8 in proteomic analysis in control and experimental treatment groups were shown in Tables 8 and 9, respectively. In control kidneys, glutathione *S*-transferase A1 and A2 and major urinary protein (Table 10) (proteins in bold letters in Table 8) were identified and were identical to those in the kidneys of the control female mice (Table 5). It is noteworthy that down-regulated glutathione S-transferase families were different between the kidney and the liver. On the other hand, in the experimental treatment kidneys, three proteins (Table 11) (proteins in bold letters in Table 9) were up-regulated, which was identical for the experimental treatment kidneys in female mice (Table 4).

## Discussion

In this study, we established a mouse model of chronic cadmium exposure. Cadmium primarily accumulated in the kidney, which is similar to findings in human chronic cadmium poisoning [3–5]. Additionally, ingested cadmium accumulated in female mice to a greater extent than in male mice, suggesting that cadmium accumulation is regulated by sex hormones, e.g., estrogen, progesterone, and testosterone. We are planning studies with castrated mouse models to test this hypothesis.

In itai-itai disease patients, cadmium is primarily accumulated in the liver probably due to impairment of cadmium storage in the disordered kidney. Complicated osteomalacia may be caused by the renal dysfunction and other pathology such as FGF23 [25], other metals, organic substances, or chemical modification of cadmium. Tissue changes in the kidney have been observed in the autopsy [7]. In experimental animals including mouse, rat, and hamster after cadmium exposure by subcutaneous injection or oral administration, renal tissue changes also have been reported [26–30]. Thijssen et al. [28] performed an experiment under similar conditions to ours and showed renal changes at an electron microscopy. The findings supported our results; there is no renal tissue change at light microscopic level.

Proteomic analysis revealed down-regulation of seven proteins including glutathione S-transferase Mu2, Mu4, and Mu7 in the liver as well as multiple proteins, including glutathione S-transferase A1 and A2, in the kidney in female and male mice after chronic oral administration of cadmium. Glutathione *S*-transferase Mu2, Mu4, Mu7, A1, and A2 are cytosolic and membrane-bound enzymes which serve as detoxifiers for electrophilic compounds. These enzymes likely are down-regulated due to overconsumption after prolonged exposure to cadmium.

On the other hand, up-regulation of two proteins in the liver and three proteins in the kidney was observed in female and male experimental groups. In the liver, peroxisomal sarcosine oxidase is up-regulated. This protein catalyzes the oxidation of the methyl group in sarcosine and the production of glycine, hydrogen peroxide, and formaldehyde. It is noteworthy that heavy metals such as cadmium inhibit this enzyme [31–33]. Based on the fact that bacterial sarcosine oxidase is induced by sarcosine [34], the eukaryotic enzyme may be also induced by excess accumulation of sarcosine.

In the kidney, a mitochondrial protein, cytochrome c oxidase subunit 6A1 is up-regulated. This protein forms one of the polypeptide chains of cytochrome c oxidase, which is the terminal oxidase in oxidative phosphorylation. Furthermore, a mitochondrial protein, cytochrome b-c1 complex subunit 2, is also up-regulated and is essential for the assembly of a cytochrome b-c1 complex, which is part of the oxidative phosphorylation cascade. It is reasonable to assume that up-regulation of these proteins induces ATP production to promote detoxification. An extracellular protein transthyretin (a carrier of the thyroid hormone thyroxine and a retinol-binding and a amyloid-related protein) is also up-regulated. However, the regulatory mechanism is unaccountable.

It has been reported that metallothionein expression is induced by acute cadmium exposure to rat [35, 36]; however, the proteomic analysis carried out in this study under chronic exposure did not identify metallothionein in either control or experimental organs, primary because mouse organs contain much fewer metallothionein (ca. 10 μg/g tissue) than other animals including human (ca. 350 μg/g in the liver and ca. 900 μg/g in the kidney) [37].

Proteomic studies related to acute cadmium poisoning have been performed on mice [20, 21] cultured rat cells [38–40], mouse cells [41, 42], and human cells [43–45], as well as other organisms [46–48]: The heat shock protein family, cytokeratin family, and Ube2d family were identified as up-regulated proteins. These findings obviously differ from the present results noted in chronic oral cadmium intake due to different pathological metabolism.

### Conclusion

Mouse model of oral intake of cadmium for chronic period (11 weeks) was established. Female and male mice took 108.8 and 115.2 μg/day/mouse, respectively as a drinking water. Cadmium storage was found predominantly in the kidney. Based on ANOVA results, cadmium was significantly accumulated in female mice more than male mice. Histological study showed that there were no changes in the kidney and the liver. Proteomic study revealed that glutathione S-transferase family was common down-regulated protein in the liver and the kidney of female and male mice.
